# Bridging the gap: Multi-sector perspectives on human, domestic animal, and wildlife leptospirosis in Ontario, Canada

**DOI:** 10.1371/journal.pone.0340404

**Published:** 2026-02-05

**Authors:** Kellie C. Libera, E. Jane Parmley, Katie M. Clow, Lauren E. Grant, J. Scott Weese, Claire M. Jardine

**Affiliations:** 1 Department of Pathobiology, Ontario Veterinary College, University of Guelph, Guelph, Ontario, Canada; 2 Department of Population Medicine, Ontario Veterinary College, University of Guelph, Guelph, Ontario, Canada; 3 Canadian Wildlife Health Cooperative, Ontario Veterinary College, University of Guelph, Guelph, Ontario, Canada; UFPL, BRAZIL

## Abstract

Although leptospirosis is one of the most common zoonotic diseases worldwide, limited surveillance and poor coordination between human and animal health sectors have resulted in scarce and disparate data on its occurrence. Strengthening integrated surveillance requires cross-sector collaboration, beginning with the engagement of key organizations. The aims of this study were to 1) determine key health experts’ awareness and risk perceptions of leptospirosis and of zoonotic disease surveillance in Ontario, Canada, and 2) examine key components of engagement, such as perceived value and interest, during the initial stages of developing an integrated leptospirosis surveillance framework. A web-based survey was sent to 543 experts in human, animal, and environmental health in Ontario, and analyzed using a mixed-methods approach to identify key factors influencing perceptions of leptospirosis, including views on *Leptospira* distribution, the impact of human behavior, and the influence of environmental conditions. Leptospirosis was recognized as a health threat in Ontario by 90% (74/82) of respondents, and 91% (70/77) indicated that current surveillance efforts are inadequate. A higher proportion of animal health sector respondents identified leptospirosis as a threat to human (93%, 37/40) and animal health (90%, 44/49) compared to public health sector respondents (76%, 25/33 and 83%, 25/30, respectively). All participants (81/81) acknowledged the benefits of integrated surveillance over the current siloed approach. Our findings highlight that key public and animal health experts perceive leptospirosis as a health threat in Ontario and support more integrated disease surveillance to better respond to this emerging zoonotic pathogen.

## Introduction

Leptospirosis, caused by pathogenic *Leptospira* spp., is one of the most prevalent zoonotic diseases worldwide, affecting both animals and humans [1,2]. Clinical manifestations vary, ranging from asymptomatic infection or mild flu-like symptoms to severe illness and death [3]. Transmission occurs primarily through indirect exposure to water or soil contaminated by the urine of infected mammals [4]. Leptospirosis is more common in tropical areas and its occurrence is heavily influenced by environmental factors, such as precipitation and temperature [5,6]. Climate change is expected to modify these factors in favour of increased *Leptospira* survival and transmission, raising health concerns even in temperate regions [7].

In North America, the apparent prevalence of leptospirosis has increased over the past two decades [3,8,9]. In 1995, leptospirosis was delisted as a nationally notifiable disease in the United States [10,11]. However, in response to more frequent outbreaks and the under-recognition of sporadic human cases, the United States reinstated leptospirosis as a nationally notifiable disease in 2014 [10,11]. Estimating the occurrence and distribution of leptospirosis in Canada is challenging due to inadequate surveillance, the absence of mandatory national reporting, and limited provincial reporting. For instance, in Ontario, leptospirosis is classified as a “periodically notifiable hazard” for animals but is not reportable in humans [12,13]. As such, leptospirosis remains under-diagnosed and understudied in Canada, as in other temperate regions [14,15], despite recently documented human and animal cases in Canada [16–18].

An integrated or One Health surveillance approach involving collaboration among public health (PH), animal health (AH), and environmental sectors has been recommended for *Leptospira* due to its environmental persistence and zoonotic potential [19–21]. For example, identification of cases in animal populations may act as an early warning for human disease, prompting timely environmental investigation and human monitoring which may improve overall detection and response [3,22]. One Health surveillance also offers broader insight into transmission dynamics and shared environmental risk factors [20,22]. However, overcoming established barriers to cross-sector coordination, including poor communication and fragmented funding, are necessary to move forward with such an approach [20].

To encourage effective collaboration across health sectors, early involvement of key experts and organizations during the development stages of integrated surveillance is crucial [20,23]. Although health experts and organizations often possess strong scientific knowledge of zoonotic diseases, their engagement in protective actions may still be influenced by subjective risk perceptions [24–26]. However, little is known about how experts across health sectors perceive the risk of leptospirosis and how these perceptions may influence surveillance efforts, particularly in temperate regions such as Ontario.

Disease risk perceptions can be examined using theoretical frameworks such as the Health Belief Model [24,27] and the Protection Motivation Theory [28]. In these frameworks, an individual’s perception of risk is influenced by both the perceived likelihood of contracting an illness and the perceived severity of its consequences [25,27,28]. Protective behaviours, such as participation in surveillance and disease mitigation programs, are more likely when individuals believe these actions are effective in reducing the threat [24,27,28].

Disease risk perception will vary based on factors such as personal beliefs, experience, and geographic location, which ultimately influences support for integrated surveillance programs [29,30]. An understanding of these perspectives can help predict stakeholder participation in integrated surveillance initiatives and contribute to more robust and inclusive health research [25,29,31]. This study aimed to assess key experts’ awareness and risk perceptions of leptospirosis, as well as their views on zoonotic disease surveillance in Ontario, Canada. The findings were contextualized using recent provincial data on leptospirosis in humans and animals from diagnostic databases and academic literature. Additionally, we examine key components of stakeholder involvement, such as perceived value and interest, during the initial stages of developing an integrated leptospirosis surveillance framework in Ontario.

## Materials & methods

### Study design

#### Study participants.

Through internet searches, personal knowledge, and personal correspondence, we identified 29 organizations in Ontario which were involved in public, animal, or environmental health and infectious disease or surveillance activities. These included, but were not limited to, federal, provincial, or local governmental departments and agencies, private diagnostic laboratories, and research groups. Through internet searches and exploration of each organization’s website, we identified individuals whose public job descriptions or job titles suggested they may work with, or may be impacted by, leptospirosis (including aspects of surveillance) in Ontario. These individuals, deemed experts, were included in the study if their contact information, such as job title, credentials, and email address were publicly available. Ethical approval for this project was granted by the University of Guelph Research Ethics Board – Natural, Physical and Engineering Sciences (REB-NPES; REB# 21-01-009). Respondents provided written informed consent through a survey response to participate anonymously in the project.

#### Survey design and data collection.

A web-based survey was developed in Qualtrics (Qualtrics, Provo, UT; S1 File). Invitations were sent via email on November 23, 2023, followed by a reminder email in January 2024. The survey remained open until January 23, 2024. In addition to capturing socio-demographic data, the survey assessed self-reported knowledge and awareness of leptospirosis and perceptions and rationales of risk posed by *Leptospira* spp., including specifics regarding risk factors in Ontario. Participants were also asked about current leptospirosis surveillance efforts and integrated surveillance for other zoonotic diseases in Ontario.

The survey included both multiple-choice and text-based questions. Participants were required to answer most questions before proceeding, except for map-based or open-ended responses. If participants indicated no knowledge of leptospirosis in either the human or animal sector, they were not asked follow-up questions on that respective topic.

#### Leptospirosis diagnostic data.

To contextualize the need for leptospirosis surveillance in Ontario, diagnostic data for human leptospirosis was provided from the Public Health Ontario (PHO) Laboratory information system on September 9, 2021, for the period of December 6, 2011 to December 31, 2020. The PHO Laboratory provides testing for individuals based on their exposure history and clinical presentation. Specimens tested for leptospirosis underwent either serological or PCR testing, dependent on the physician’s request. Ethical approval for obtaining PHO diagnostic data was granted by the University of Guelph REB-NPES, REB# 21-06-012. Animal diagnostic data for the same period were obtained from the Animal Health Laboratory (AHL) in Guelph, where serological testing using MAT was performed for the following serovars: Autumnalis, Bratislava, Canicola, Grippotyphosa, Hardjo, Icterohaemorrhagiae and Pomona. Both PHO and AHL define seropositivity as a titre ≥1:100. No identifying information, including clinical data or vaccination history, were obtained to maintain patient anonymity.

### Data analysis

#### Descriptive statistics.

**Fig 1:**
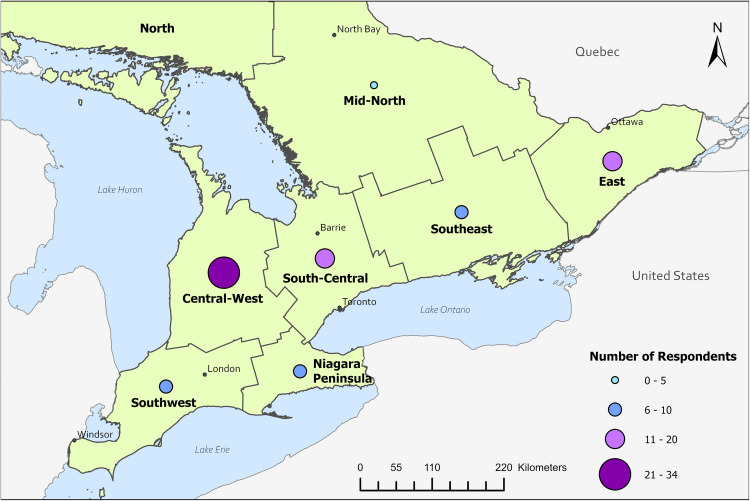
Employment location of study participants in Ontario. The nine regions are based on employer Forward Sortation Area in Ontario (N = 101; eight participants were employed in a province outside of Ontario). Base maps were reproduced under the Statistics Canada Open License and U.S. Census Bureau public domain [32–34].

**Fig 2:**
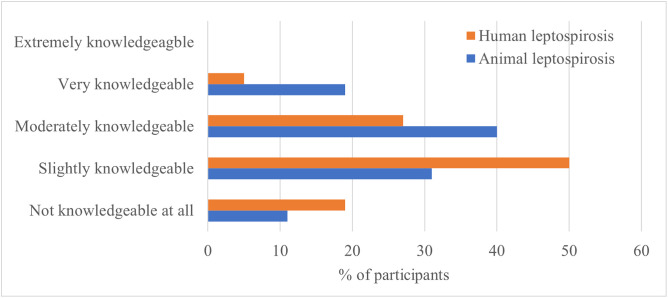
Self-assessed knowledge level of human and animal leptospirosis from health professionals in Ontario (N = 101).

**Fig 3:**
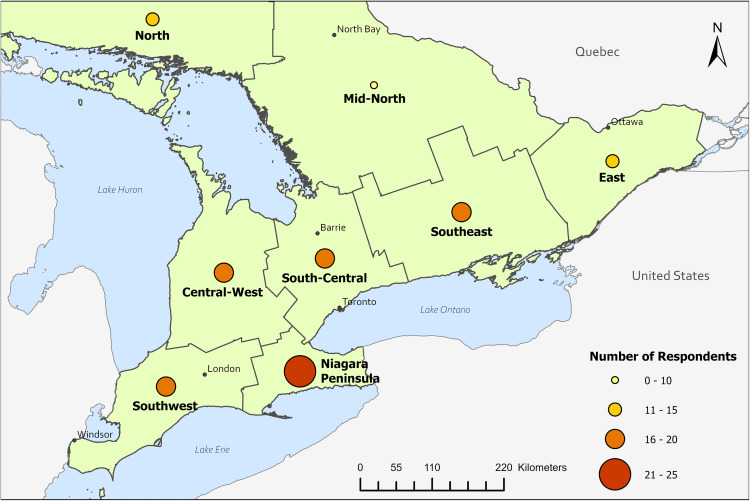
Regions in Ontario identified by respondents as having leptospirosis activity (n = 122 datapoints from 40 respondents). 78 participants responded to the question. 40 participants selected at least one region, 38 were unsure and are not represented on this map. Base maps were reproduced under the Statistics Canada Open License and U.S. Census Bureau public domain [32–34].

Survey data were summarized using Qualtrics and Excel (Figs 1–3, Tables 1–4, S1–S5 Tables). Response percentages (%, n/N) and 95% confidence intervals [CI] were calculated in Excel using the Wilson interval score with continuity correction to account for proportions near 1 or small sample sizes. Ontario was divided into eight regions, as per the Ontario Ministry of Public and Business Service Delivery [35], and participant location was categorized within these regions based on employer Forward Sortation Area. Maps were created using ArcGIS Pro (version 3.3) and were adapted from boundary files available from Statistics Canada (Statistics Canada Open License) and U.S. Census Bureau (public domain) [32–34].

#### Statistical analysis.

Participants were categorized into public health or animal health sectors based on their educational background, or current employment role if background information was not provided. For participants with interests in both sectors, they were categorized into the sector in which they were more involved, typically based on their background training. There were no individuals that were trained in one field and worked solely in the other, although it is possible individuals may work in roles that incorporate both fields. Comparisons between the two sectors were made using a Chi-squared test with a significance value of α = 0.05. Due to varying responses rates across questions, the z8 statistic for two partially overlapping samples, which include both independent and paired samples, derived from a one-sample proportion test (‘prop.test’ in the ‘partiallyoverlapping’ R package, version 4.1.1) was used to compare perceptions of leptospirosis risk across different populations (humans, domestic animals, and wildlife). The z8 test statistic assumes equal participation for calculations within the package and effect size was estimated using Cohen’s *h* ($h=\text{2}\;x\;arcsin\sqrt{p\text{1}}-arcsin\sqrt{p\text{2}}$) and interpreted with the following scale: negligible *h* < 0, small 0.20 ≤ *h* < 0.50, medium 0.50 ≤ *h* < 0.80, large *h* ≥ 0.80 [36,37].

#### Qualitative analysis.

Qualitative responses were coded and summarized using Excel and Word, applying an interpretive approach and thematic analysis. This methodology was based on guidance from Green and Thorogood [31] and similar studies [38–40]. The initial analysis was performed by KCL, followed by review and discussion with CMJ and EJP to refine the final interpretation. While many themes were identified a priori using a deductive approach (i.e., a codebook), inductive methods (i.e., open coding) were also applied to identify emergent themes or subthemes. These included motivations and reasons for responses, such as explicit knowledge (e.g., “*Recent studies have demonstrated…*”), opinion or anecdote (e.g., “*I have seen clinical cases…*”), and uncertainty (e.g., “*I would suspect so but I am unsure*”). Any reorganization of a priori themes and creation of new categories was carried out by KCL early in the analysis. Subthemes emerged during the process, with responses potentially fitting under multiple themes or subthemes depending on context. Relevant quotes from respondents are provided to illustrate the findings.

## Results

### Participant characteristics

Eight-hundred individuals were identified as potential participants. Of these, 543 had publicly available email addresses and were contacted to participate in our survey. After excluding 24 undeliverable emails and 7 out-of-office replies, the effective sample size was 512 individuals, and the survey response rate was 21% (109/512). The number of responses (N) varied across questions due to incomplete surveys or questions not presented based on some respondents' previous responses or occupation. The most often reported age category was 40–49 years old (32%, 34/108), and most respondents identified as women (74%, 80/108). Among key experts, 77% (59/77) expressed interest in receiving study results, and 60% (46/77) indicated a willingness to assist with the next stages of the project. Geographically, participants were most frequently employed in the Central-West (33%, 34/102), South-Central (17%, 17/102), and East (17%, 17/102) regions of Ontario (Fig 1).

There was balanced representation from the public health (PH; 44%, 47/107) and animal health (AH; 56%, 60/107) sectors (χ^2^ = 1.35, p = 0.25), with participants representing all organizational levels (Table 1). The most common areas of employment or advanced training were veterinary medicine (49%, 53/108), epidemiology (31%, 33/108), biology (17%, 19/108), and human medicine (13%, 14/108). Notably, 43% (14/33) of epidemiologists were also trained as veterinarians (S1 Table). At the time of the survey, 58% (60/104) were in their current role for six or more years, 37% (38/104) for over ten years, and 42% (36/85) reported current involvement with *Leptospira*.

**Table 1:** Employment organizations of public and animal health sector respondents and organizational level of these government and non-government agencies.^a^

Organization level	Employment organizations of public health sector experts (n = 47)	Employment organizations of animal health sector experts (n = 60)
Federal government (n = 52)	• Public Health Agency of Canada • Environment and Climate Change Canada • Canadian Food Inspection Agency	• Public Health Agency of Canada • Environment and Climate Change Canada • Canadian Food Inspection Agency • Parks Canada
Provincial government (n = 17)	• Ontario Health • Ontario Ministry of the Environment, Conservation and Parks • Ontario Ministry of Health • Public Health Ontario	• Ontario Ministry of Agriculture, Food and Rural Affairs • Ontario Ministry of Natural Resources and Forestry
County or city (n = 14)	• Public health unit • City	• Public health unit • City
Academia (n = 17)	• University, college, or other educational institution	• University, college, or other educational institution
Other: non-government or private organizations (n = 13)	• Hospital • Diagnostic laboratory	• Veterinary clinic • Diagnostic laboratory

^a^The number of responses for employment location was N = 107 and N = 102 for organization level. Some participants may not have responded to both questions or may have had more than one employer within the same or different organizational levels.

### Self-reported leptospirosis knowledge level

Most respondents indicated being at least slightly knowledgeable about both animal and human leptospirosis (76%, 77/101), 88% (89/101) about animal leptospirosis, and 81% (82/101) about human leptospirosis. Nineteen percent of respondents (19/101) reported being very knowledgeable about animal leptospirosis, with the majority belonging to the AH sectors. Only five percent (5/101), all from the PH sector, reported similar knowledge of human leptospirosis (S2 Table). No participants reported being extremely knowledgeable about either population (Fig 2).

### Leptospirosis risk in Ontario

#### Perceived risk.

Most experts perceived leptospirosis as a health risk in Ontario (90%, 74/82), specifically for humans (85%, 62/73) and domestic animals (87%, 69/79; Table 2), with no difference in risk between these two populations (p = 0.452; S3 Table). Although not statistically significant, experts from the AH sector were more likely to perceive leptospirosis as a risk in Ontario compared to those from the PH sector (96%, 47/49 vs 82%, 27/33, p = 0.055; S4 Table). This difference was most notable within the moderately knowledgeable category, where 67% (6/9) from the PH sector viewed a human health risk compared to 92% (12/13) from the AH sector, and where 75% (6/8) from the PH sector viewed an animal health risk compared to 92% (23/25) from the AH sector (S2 Table). Experts viewed a significantly lower risk to wildlife health compared to humans and domestic animals (p < 0.001; S3 Table).

**Table 2:** Perceived health risk, impacts of concern, and surveillance adequacy for leptospirosis in Ontario.^a, b^

Question category	Response options	% Participants (n)
Leptospirosis perceived as a health risk	Ontario overall (N = 82)	90 (74)
	Domestic animal health (N = 79)	87 (69)
	Human health (N = 73)	85 (62)
	Wildlife health (N = 79)	54 (43)
Impacts of concern^c^ (N = 79)	Domestic animal health	
	• clinical disease • death• long-term effects	53 (42)15 (12)8 (6)
	Human health	
	• clinical disease • death • long-term effects	58 (46) 13 (10) 19 (15)
Wildlife illness or mortality	10 (8)
Economic consequences	29 (23)
Leptospirosis surveillance perceived as adequate (N = 77)	Animals only	9 (7)
	Humans only	14 (11)
	Both humans and animals	9 (7)
	Neither humans or animals	68 (52)

^a^A list of responses was presented to respondents in multiple choice format.

^b^Further analysis of data by health sector is presented in S4 Table.

^c^Participants were able to choose up to three impacts they perceived as concerning.

Common issues affecting disease risk perception included clinical disease in humans (58%, 46/79) and in domestic animals, including livestock (53%, 42/79), along with economic consequences (29%, 23/79; Table 2). However, fewer participants from the PH sector identified these concerns compared to the AH sector (S4 Table).

#### Qualitative responses: justification for risk perception.

Responses provided to justify a participant’s perception of leptospirosis risk for humans, domestic animals, and wildlife health were categorized into six themes: disease epidemiology, environmental risk factors, host risk factors, pathogen risk factors, perception and awareness, and prevention and control measures (Table 3).

**Table 3:** Summary of the described themes from the qualitative analysis of experts’ rationale for perceived leptospirosis health risk in Ontario. Themes and subthemes were derived from text-based survey responses in which experts justified their perception for or against leptospirosis as a health risk for humans, domestic animals, and wildlife in Ontario. The number of respondents who referenced each theme and/or subtheme are indicated for each population category and further categorized by the participant's perception of a health risk to that population (human, domestic animal, or wildlife). Responses may fall into more than one theme or subtheme, which may cause discrepancies between the sum of subtheme counts and the total listed for the main theme.

Themes	Sub-themes	Further description and examples	Is there a health risk to…					
			humans?		domestic animals?		wildlife?	
			Yes n = 60	No n = 11	Yes n = 68	No n = 8	Yes n = 34	No n = 33
Disease epidemiology			32	4	25	1	10	12
	Disease ecology	Ecological succession; scale of impact (individual vs population); ubiquitous or endemic nature of leptospirosis	8		4		2	3
	Incidence	Reference to outbreaks or increasing cases; presence of pathogen in Ontario	8	1	7		1	1
	Reservoirs	Domestic animals or wildlife as carriers or reservoirs of disease; presence of disease reservoirs in Ontario	11	1	5	1	4	7
	Risk	Changing risk (increasing or decreasing); level of risk (none, low, high); future risk; comparing risk to other diseases, species, or locations	11	2	6		4	2
	Transmission	Transmission by animals; shedding; not transmissible in humans	9	1	5		1	
Environmental factors			24	4	26		4	1
	Climate change	May influence disease risk, migration patterns, or weather events such as flooding	4				2	
	Exposure to contaminated environments	Exposure to water, wildlife, pets, livestock, or geographic area	20	1	26		2	1
	Suitability of environmental conditions	Environmental conduciveness to pathogen survival; temperate (e.g., Ontario) vs tropical locations	1	3	1			
Host factors			12		19		15	13
	Behavioural	Travel (of human, domestic animal, or wildlife); occupation or recreation (of human or domestic animal)	5		4			
	Disease impact or severity	No clinical disease/asymptomatic; potential for clinical or subclinical effects; mild illness or self-limiting disease; serve illness or mortality	8		6		14	13
	Species specific susceptibility	Certain domestic animals or wildlife may be more susceptible to disease			9		9	3
Pathogen factors		Reference to zoonosis, infectious or contagious disease, or serovars	20		4		3	
Perception and awareness			18	3	13	3	19	23
	Lack of public awareness	Lack of public awareness or media attention; inadvertent exposure due to low awareness	6		1			
	Participant uncertainty; limited information	E.g., *“Not sure”, “I believe…”, “I assume…”*; discussion of hard to track cases; information or evidence not available or accessible	6	1	2	3	18	22
	Personal or professional experience	Reference to direct personal or professional experience or knowledge; under diagnosis and under reporting	8	2	10		1	1
Prevention and control measures			4	1	13	5		
	Effective control measures	Reference to disease control, effective vaccination, or treatment; proper precautions taken	1	1	5	5		
	Limitations or lack of biosecurity	Limitations or lack of vaccination, hand washing, PPE; ineffective precautions	3		8			

*Disease epidemiology:* Epidemiological concepts, such as disease occurrence, transmission, and ecology, were referenced the most when participants explained their perception of risk regarding human health (51%, 36/71), but less in the context of domestic animal health (35%, 26/74). Within the subthemes of both incidence and risk, some participants emphasized that low incidence or low risk does not fully negate potential risk. However, a few participants argued the converse that low incidence did correlate with low or no risk. Several respondents compared perceived risks relative to other species, locations, or diseases.

*Disease risk factors:* Among participants who identified a human health and/or domestic animal health risk, environmental factors were referenced most frequently to explain this stance (40%, 24/60 and 38%, 26/68, respectively), with particular emphasis on exposure to contaminated pets and environments. In contrast, host factors were referenced most frequently with respect to wildlife (42%, 28/67) and used to justify both a perceived presence and absence of health risk from leptospirosis. A greater proportion of respondents referenced pathogen factors as a reason for perceiving a risk to human health (28%, 20/71) compared to domestic animal (5%, 4/74) or wildlife health (5%, 3/67).

*Perception and awareness:* This was the most prominent theme noted regarding leptospirosis impacts on wildlife (63%, 42/67), with over half of respondents referencing the subtheme of uncertainty (60%, 40/67). In contrast, expressions of uncertainty were far less common for human health (10%, 7/71) and domestic animal health (5%, 5/74).

*Prevention and control measures:* Some participants perceived the availability of vaccines and treatments as mitigating risks, while others viewed the existence of vaccines as indicative of a potential risk. Vaccination was referenced nearly four times more frequently in responses concerning domestic animal health (n = 15) than human health (n = 4).

#### Additional risk factor information.

When provided with a list of risk factors, the most frequently selected for humans were host behavior (30%, 21/70) and contact with domestic and agricultural animals (25%, 18/70; Table 4). For companion animals, host behaviour was also the most common (35%, 28/79), followed by geographic location, land use, and/or habitat (33%, 26/79). The locations in Ontario that were most commonly perceived as having leptospirosis activity included the Niagara Peninsula (58%, 23/40), South-Central (48%, 19/40), South-West (48%, 19/40), Central-West (45%, 18/40), and South-East (43%, 17/40) regions (Fig 3).

**Table 4:** Key risk factors for leptospirosis in humans, companion animals, and livestock in Ontario, as identified by participants.^a^

Leptospirosis risk factor	Population category (% participants (n))		
	Humans (N = 70)	Companion animals (N = 79)	Livestock (N = 79)
Contact with domestic or agriculture animals	26 (18)	5 (4)	14 (11)
Contact with wildlife	16 (11)	23 (18)	34 (27)
Geographic location, land use and/or habitat	14 (10)	33 (26)	28 (22)
Host (animal) behaviour	N/A	35 (28)	6 (5)
Host (human) behaviour	30 (21)	N/A	N/A

N = total number of participants who responded to the question; n = number of participants who perceived the factor as a risk for leptospirosis.

^a^Participants were able to select up to three risk factors. Additional risk factors with less than 11% responses were not included in table (e.g., weather or climate, socio-demographic factors, other).

### Surveillance perceptions

Most respondents (91%, 70/77) indicated that current leptospirosis surveillance in Ontario is inadequate for humans, animals, or both (Table 2). Slightly more respondents perceived surveillance as inadequate for humans (82%, 63/77) than for animals (77%, 59/77). All respondents (100%, 81/81) agreed that cross-sector involvement and data integration would improve zoonotic disease surveillance.

### Leptospirosis cases in Ontario

Between December, 2011 and December, 2020, PHO documented 83 positive and 24 inconclusive tests for humans. During the same period, the AHL documented 7046 serologically positive tests among domestic, wildlife, and zoo animals; bovine (n = 2999) and canine (n = 2275) had the most seropositive results (S5 Table).

## Discussion

Experts from both the public health (PH) and animal health (AH) sectors recognize leptospirosis as a health risk in Ontario. Interestingly, a greater proportion of AH experts perceived leptospirosis as a human health risk compared with PH experts. This difference may reflect the higher self-reported knowledge of leptospirosis among AH participants, which could contribute to greater awareness of zoonotic transmission pathways and associated human exposure risks. Veterinarians, who comprised approximately 50% of AH participants, may contribute more to this perspective due to their training and professional experience with zoonotic disease [41]. In addition, leptospirosis case counts were much lower in humans compared to animals in Ontario. This may have further limited PH professional exposure to leptospirosis, and therefore reduced awareness overall. A similar lack of professional exposure, contributing to a knowledge and diagnostic gaps, has been observed in other North American regions where leptospirosis incidence is increasing [14].

Many respondents noted that a lack of accessible information on human and wildlife leptospirosis may have affected their perception of risk. In the absence of reliable data, individuals may either underestimate risk due to limited public health messaging, or overestimate risk if they are risk-averse and uncertain about actual exposure [42]. Risk aversion should not be underestimated in terms of shaping perception, even in the face of expert knowledge. For instance, experts who were knowledgeable about leptospirosis vaccination recommendations and efficacy were divided in their perception of leptospirosis as a health risk. For risk adverse individuals, awareness that a pathogen exists at levels high enough to warrant vaccination recommendations may be more influential on risk perception than the knowledge of vaccination efficacy. As one participant wrote, “*A pathogen that is present in the environment and can affect humans is a public health risk; it doesn’t have to be common to be a risk.”* This further highlights the importance of integrative communication strategies that contextualize risk and promote consistent messaging [42].

All experts were less familiar with leptospirosis in wildlife but did perceive a lesser health impact on wild species than on humans or domestic animals. This coincides with the current view that both the clinical impact on wildlife and their role in *Leptospira* transmission are poorly understood [43].

Globally, health experts have expressed high levels of support for One Health approaches to mitigate neglected tropical diseases, including leptospirosis, with surveys reporting up to 98% agreement and no significant differences between human and animal health sectors [38,44]. Respondents in our study similarly viewed existing siloed approaches as insufficient and unanimously agreed that an integrated framework would be more effective.

Regarding location, participants perceived *Leptospira* activity across Ontario, but recognized warmer, southern regions as having higher risk. This aligns with a recent Ontario study that identified multiple localized areas within these regions as potential hotspots for canine infections [17], suggesting that areas of overlap may be suitable for targeted integrated surveillance pilot studies. Experts often identified their own districts as areas of active leptospirosis transmission, even when this perception was not shared by participants outside those regions, indicating that leptospirosis activity may be more widespread than currently recognized. This pattern highlights the importance of both actual and perceived local knowledge in informing larger scale surveillance efforts since regional insights and drivers of disease, such as access to healthcare and diagnostic services, may not always be effectively communicated at the provincial level [20,45,46]. Greater disease awareness in high-risk areas is expected, particularly for diseases associated with severe clinical outcomes [25,42]. Because higher perceived risk is more likely to motivate participation, understanding the factors that shape perception is essential for sustained engagement and long-term surveillance success [28,29].

In agreement with recent literature, participants identified environmental contamination and human-animal interactions as key factors influencing their perception of leptospirosis risk [5]. These exposure pathways are well-established drivers of leptospirosis transmission across human, animal, and environmental interfaces [47]. However, while written responses emphasized zoonotic transmission and environmental exposure, multiple-choice selections more often prioritized host behavior as a primary risk factor. Experts may view contaminated environments as an inherent aspect of leptospirosis endemicity, and as a result, may prioritize behaviour as a risk factor, recognizing that avoiding exposure is challenging [17]. One participant summarized this perspective: *“*Leptospira *can be transmitted through a variety of sources, including wildlife, environmental water/soil, and domestic animals. Those with higher contact with any of these sources are at risk. Due to lack of knowledge and reporting, individuals likely are not taking the necessary precautions to reduce infection risk.”*

As with similar qualitative studies, not all findings in our study aligned with the predefined themes, requiring inductive theme creation during analysis [40]. Although we did not assess the underlying sources or motivations behind experts’ knowledge and opinions, there is likely a gap between perceived and actual knowledge. For example, some participants provided treatment and vaccination information that was not in line with current Ontario human or veterinary medical association recommendations. Addressing specific disease and risk knowledge gaps as part of a pilot program could enhance long-term participation and compliance for integrated surveillance.

**Fig 4:**
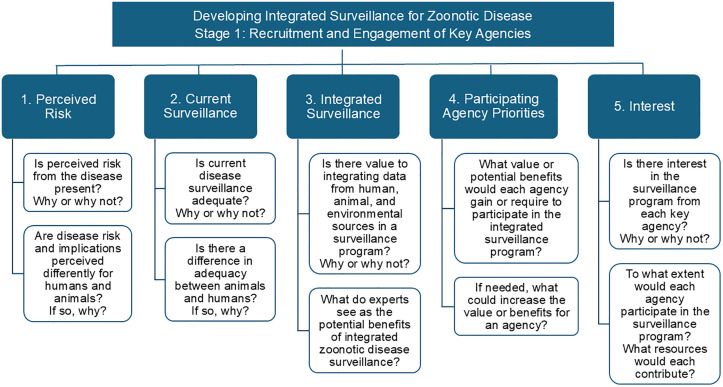
Expanded framework for the initial stages of developing an integrated surveillance program for zoonotic disease. We have identified five key subcomponents for the initial stakeholder recruitment and engagement stage of integrative surveillance development based upon the structural frameworks proposed by OHHLEP et al. [20] and Ghai et al. [23]. For each component, we have suggested questions to guide researchers to engage and recruit key agencies.

The importance of engaging stakeholders early in the development of One Health surveillance systems is well recognized and several frameworks have proposed multi-stage processes, with initial stages focused on stakeholder identification and engagement, along with defining current policy and the scope of the initiative [20,23]. Our study was aligned with the first stage of these frameworks, prioritizing the identification of stakeholder interest and feasibility of leptospirosis disease surveillance. Building upon these established models, we propose a refinement of this initial phase to assess stakeholder readiness, motivations, and long-term engagement potential (Fig 4). This expanded approach may support more resilient participatory surveillance systems, particularly for complex, environmentally mediated zoonoses such as leptospirosis.

As participatory research methods offer valuable insights into the diverse priorities of key actors [48], experts from both the AH and PH sectors were recruited, with response rates comparable to similar studies [38,49]. However, difficulties in accessing contact information for private organizations, such as laboratories and clinics, may have resulted in under sampling from these organizations. Low private sector participation is a recognized barrier to disease surveillance [50]. Identifying the needs and priorities of these organizations could benefit surveillance efforts and facilitate funding, which remains a significant challenge to endemic disease monitoring [23,38]. This is particularly important in Ontario as a notable portion of leptospirosis diagnostics are performed by private laboratories (e.g., IDEXX, LifeLabs). Successful private sector integration into surveillance programs in countries like France and Germany may provide useful models for North American efforts [51].

In Ontario, vaccination against leptospirosis is generally offered as a risk-based vaccine rather than part of core vaccine schedules for companion animals (dogs) and livestock (cattle, pigs, horses), and province-wide vaccination coverage is not reported [52,53]. There is no vaccine licensed for use in humans in Canada [15]. Recently, the American Animal Hospital Association (AAHA) updated its core vaccination recommendations for dogs to include leptospirosis in endemic areas [54]. As many Ontario clinics are AAHA accredited, more clinics may adopt this policy. We did not have information about the immunization status of the animals tested and this information gap must be considered when interpreting serological results.

An additional limitation was that online surveys are subject to response and self-selection bias, and purposive sampling may limit the generalizability of findings to larger populations [55]. For instance, categorical responses may misrepresent participants’ opinions due to biases such as acquiescence and question order effects [56]. We presented open-ended questions prior to related multiple-choice questions to avoid pre-conditioned responses and did not make populations level inferences to help mitigate these effects. The increasing recognition of qualitative and descriptive research in science underscores the importance of contextual knowledge to obtain a more complete understanding of a given issue [31]. Benefits of using different data collection methods were reinforced in this study as our qualitative and quantitative approaches yielded different results for similar questions (e.g., the rationale for participants' perception of leptospirosis risk).

To advance One Health leptospirosis surveillance development for Ontario, future research should aim to increase private-sector, humanities, and Indigenous community participation, evaluate current data collection practices, and map communication pathways between key organizations [46,47,52,57]. Strengthening surveillance efforts will require exploring the implications of designating leptospirosis as a reportable disease and integrating elements from successful One Health programs in Canada.

The early engagement of experts from key organizations is essential for the successful development and long-term sustainability of an integrated One Health surveillance program [46,57]. However, such engagement is rarely documented. Experts from both Ontario’s AH and PH sectors agree that leptospirosis poses a current health risk to animals and humans and that current surveillance efforts are inadequate. They agree that an integrated surveillance approach is preferred for monitoring zoonotic diseases and also showed interest in future research findings. Understanding perceptions of leptospirosis risk in Ontario can inform more effective recruitment and targeted educational efforts, supporting sustained participation surveillance initiatives. The early involvement of key public and animal health experts in this study highlights the feasibility of engaging stakeholders and the potential value of an integrated approach to leptospirosis surveillance in Ontario.

## Supporting information

S1 Table Educational background and professional training of participants. (DOCX)

S2 Table Comparison of self-reported leptospirosis knowledge by expert health sector and perceived health risk. (DOCX)

S3 Table Comparison of leptospirosis risk perception for different populations within Ontario. (DOCX)

S4 Table Further analysis by health sector of perceived health risk, impacts of concern, and surveillance adequacy of leptospirosis in Ontario. (DOCX)

S5 Table Seropositive leptospirosis MAT results for domestic animals, zoo animals, and wildlife from the Animal Health Laboratory in Guelph, Ontario (December, 2011 to December, 2020). (DOCX)

S1 File Survey questions. (DOCX)
