# Supplementary material for: Bridging the gap: Multi-sector perspectives on human, domestic animal, and wildlife leptospirosis in Ontario, Canada
Source: PLoS One. 2026 Feb 5;21(2):e0340404. doi: 10.1371/journal.pone.0340404 (PMC12875493; doi:10.1371/journal.pone.0340404)
Supplement: S1 Table — (DOCX) [file pone.0340404.s001.docx]

**S1 Table. Educational background and professional training of participants** (N=108). ^a^

| **Educational background or training** | **% Respondents** (n) | **Secondary employment information** ^b^ | **% Respondents** (n) |
| --- | --- | --- | --- |
| Biologist | 18 (19) | Microbiologist | 1 (1) |
|  |  | Business or management | 1 (1) |
| Business or management | 5 (5) |  |  |
| Epidemiologist | 31 (33) | Veterinarian | 13 (14) |
|  |  | Human physician | 2 (2) |
|  |  | Academia/professor | 6 (6) |
|  |  | Business or management | 1 (1) |
|  |  | Biologist | 6 (6) |
| Human physician (MD or equivalent) | 13 (14) | Academia/professor | 7 (8) |
|  |  | Government | 2 (2) |
|  |  | Public health and/or preventive medicine | 5 (5) |
| Laboratory technician | 1 (1) |  |  |
| Professor | 7 (7) |  |  |
| Veterinarian (DVM or equivalent) | 49 (53) | Academia/professor | 7 (7) |
|  |  | Small animal practice | 3 (3) |
|  |  | Biologist | 2 (2) |
|  |  | Mixed practice | 1 (1) |
|  |  | Government | 39 (42) |
|  |  | Referral or specialty center | 1 (1) |
|  |  | Private diagnostics | 1 (1) |
|  |  | Pathologist | 1 (1) |
| Nurse | 2 (2) | Registered nurse | 1 (1) |
|  |  | Public health nurse | 1 (1) |
| Other: Public health | 7 (8) | Public health inspector/supervisor | 1 (1) |

^a^ Participants may have identified with more than one educational field, specialty, or employment category

^b^ Participants had the option to give information about a secondary field of expertise, training, or specialty within their primary field.
