# Supplementary material for: Bridging the gap: Multi-sector perspectives on human, domestic animal, and wildlife leptospirosis in Ontario, Canada
Source: PLoS One. 2026 Feb 5;21(2):e0340404. doi: 10.1371/journal.pone.0340404 (PMC12875493; doi:10.1371/journal.pone.0340404)
Supplement: S2 Table — (DOCX) [file pone.0340404.s002.docx]

**S2 Table . Comparison of self-reported leptospirosis knowledge by health sector and perceived health risk. ^a^**

| **Knowledge level** | **Participants who perceived a risk to human health ^b^** | **Respondents who perceived a risk to animal health ^c^** |
| --- | --- | --- |
|  | % (n/N) | % (n/N) |
| Slightly knowledgeable |  |  |
| All | 88 (38/43) | 86 (24/28) |
| Public health experts | 81 (13/16) | 86 (18/21) |
| Animal health experts | 93 (25/27) | 86 (6/7) |
| Moderately knowledgeable |  |  |
| All | 82 (18/22) | 88 (29/33) |
| Public health experts | 67 (6/9) | 75 (6/8 |
| Animal health experts | 92 (12/13) | 92 (23/25) |
| Very knowledgeable |  |  |
| All | 80 (4/5) | 89 (16/18) |
| Public health experts | 80 (4/5) | 100 (1/1) |
| Animal health experts | 0 | 88 (15/17) |
| Extremely knowledgeable |  |  |
| All | 0 | 0 |
| Public health experts | 0 | 0 |
| Animal health experts | 0 | 0 |

^a^ Those who selected ‘not knowledgeable at all’ or did not provide perceptions regarding both human and animal health risk are not included in this table.

^b^ 86% (60/70) respondents perceived a risk to human health, 38% (23/60) were public health experts and 62% (37/60) were animal health experts.

^c^ 87% (69/79) respondents perceived a risk to animal health, 36% (25/69) were public health experts and 64% (44/69) were animal health experts.
