# Supplementary material for: Bridging the gap: Multi-sector perspectives on human, domestic animal, and wildlife leptospirosis in Ontario, Canada
Source: PLoS One. 2026 Feb 5;21(2):e0340404. doi: 10.1371/journal.pone.0340404 (PMC12875493; doi:10.1371/journal.pone.0340404)
Supplement: S3 Table — (DOCX) [file pone.0340404.s003.docx]

**S3 Table. Comparison of leptospirosis risk perception for different populations within Ontario**. ^a^

| **Population risk category** | **Domestic animal health** | **Public health** | **Wildlife health** |
| --- | --- | --- | --- |
| **All of Ontario** | z8 = -1.1436 | z8 = -2.0166 | z8 = 6.1256 |
|  | p = 0.253 | p = 0.044***** | p = <0.001***** |
|  | *h =* 0.0922 - small | *h* = 0.140 – small | *h* = 0.8467 - large |
|  | *h* 95% CI = -0.217, 0.401 | *h* 95% CI = -0.217, 0.401 | *h* 95% CI = 0.538, 1.156 |
| **Domestic animal health** |  | z8 = 0.7517 | z8 = 4.8272 |
|  |  | p = 0.452 | p = <0.001***** |
|  |  | *h* = 0.048 – small | *h* = 0.7546 – medium |
|  |  | *h* 95% CI = -0.274, 0.370 | *h* 95% CI = 0.443, 1.066 |
| **Public health** |  |  | z8 = 4.3574 |
|  |  |  | p = <0.001* |
|  |  |  | *h* = 0.72 – medium |
|  |  |  | *h* 95% CI = 0.402, 1.045 |

Four population health risk categories (all of Ontario, domestic animals, wildlife, and humans) were compared using the z8 statistic and Cohen’s *h* effect size.

^a^ CI = *h*±z_α/2_​⋅SE(*h*)
