# Supplementary material for: Bridging the gap: Multi-sector perspectives on human, domestic animal, and wildlife leptospirosis in Ontario, Canada
Source: PLoS One. 2026 Feb 5;21(2):e0340404. doi: 10.1371/journal.pone.0340404 (PMC12875493; doi:10.1371/journal.pone.0340404)
Supplement: S4 Table — (DOCX) [file pone.0340404.s004.docx]

**S4 Table. Further analysis by health sector of perceived health risk, impacts of concern, and surveillance adequacy of leptospirosis in Ontario.**

| **Population category** | **All participants** | **Participants from public health sector** | **Participants from animal health sector** | **Fisher’s exact ^a^** |
| --- | --- | --- | --- | --- |
| **Perceived health risk ^b^** | % [95% CI]; n/N | % [95% CI]; n/N | % [95% CI]; n/N | p-value |
| Ontario health overall | 90 [81-95]; 74/82 | 82 [64-93]; 27/33 | 96 [85-99]; 47/49 | 0.055 |
| Human health | 85 [74-92]; 62/73 | 76 [58-88]; 25/33 | 93 [79-98]; 37/40 | 0.057 |
| Domestic animal health | 87 [77-93]; 69/79 | 83 [64-93]; 25/30 | 90 [77-96]; 44/49 | 0.492 |
| Wildlife health | 54 [42-65]; 43/79 | 50 [32-68]; 15/30 | 57 [42-71]; 28/49 | 0.643 |
| **Impacts of concern** | % (n); N=79 | % (n); N=35 | % (n); N=44 |  |
| Humans |  |  |  |  |
| - Clinical disease | 58 (46) | 51 (18) | 64 (28) |  |
| - Death | 13 (10) | 17 (6) | 9 (4) |  |
| - Long-term clinical effects | 10 (15) | 20 (7) | 18 (8) |  |
| Domestic animals |  |  |  |  |
| - Clinical disease | 53 (42) | 43 (15) | 61 (27) |  |
| - Death | 15 (12) | 9 (3) | 20 (9) |  |
| - Long-term clinical effects | 8 (6) | 9 (3) | 7 (3) |  |
| Wildlife |  |  |  |  |
| - Illness or mortality | 10 (8) | 14 (5) | 7 (3) |  |
| All |  |  |  |  |
| - Economic consequences | 29 (23) | 20 (7) | 36 (16) |  |
| - Uncertain or other | 3 (4) | 3 (1) | 5 (2) |  |
| **Is current surveillance adequate?** | % (n); N=77 | % (n); N=32 | % (n); N=45 |  |
| Yes | 9 (7) | 6 (2) | 11 (5) |  |
| Yes (humans only) | 9 (7) | 19 (6) | 2 (1) |  |
| Yes (animals only) | 14 (11) | 16 (5) | 13 (6) |  |
| No | 68 (52) | 59 (19) | 73 (33) |  |

^a^ Fisher’s exact comparison is between participants from the public health sector and participants from the animal health sector.

^b^ Results in this table represent participants who perceived that there is a health risk from leptospirosis for that respective population category.
