# Supplementary material for: Bridging the gap: Multi-sector perspectives on human, domestic animal, and wildlife leptospirosis in Ontario, Canada
Source: PLoS One. 2026 Feb 5;21(2):e0340404. doi: 10.1371/journal.pone.0340404 (PMC12875493; doi:10.1371/journal.pone.0340404)
Supplement: S5 Table — (DOCX) [file pone.0340404.s005.docx]

**S5 Table. Seropositive leptospirosis MAT results for domestic animals, zoo animals, and wildlife from the Animal Health Laboratory in Guelph, Ontario (December, 2011 to December, 2020).** ^a,b^

| **Family/Order** | **Number of seropositive tests (**titre ≥1:100) |
| --- | --- |
| Family Bovidae | 3021 |
| Family Camelidae | 11 |
| Family Canidae | 2275 |
| Family Equidae | 684 |
| Family Suidae | 961 |
| other Order Carnivora | 51 |
| other | 22 |

^a^ Individual animals may have been tested more than one time.

^b^ Results are based on test report dates, not sample collection dates. Serum samples may have been frozen and stored, thus collected before 2011 and reported here, or collected during our reporting period, stored and tested after our reporting period.
