# Supplementary material for: Bridging the gap: Multi-sector perspectives on human, domestic animal, and wildlife leptospirosis in Ontario, Canada
Source: PLoS One. 2026 Feb 5;21(2):e0340404. doi: 10.1371/journal.pone.0340404 (PMC12875493; doi:10.1371/journal.pone.0340404)
Supplement: S1 File — (DOCX) [file pone.0340404.s006.docx]

**S1 File. Survey Questions ^a^**

**Demographics**

1. Please select your age category: *18-29 years old; 30-39 years old; 40-49 years old; 50-59 years old; 60-69 years old; 70-79 years old; >80 years old; Prefer not to answer*
2. How would you describe your gender identity? *Agender; Gender fluid; Gender queer; Woman (includes cis-women, trans-women, and everyone else who identifies as a woman); Non-binary; Man (includes cis-men, trans-men, and everyone else who identifies as a man); Transgender; Prefer to self-describe _____; Prefer not to answer*
3. What is your educational background and training? Select all that apply: *Biologist; Business/management (any); Epidemiologist; Human physician (MD or equivalent); Laboratory technician; Professor; Research/field technician; Veterinarian (DVM or equivalent); Other _____*
4. First 3 digits of employer postal code (e.g., J8P). If you work in >1 location, enter most frequent location: _____
5. Name of current employer(s): Please select as many as apply: *Animal Health Laboratory (Guelph); Canadian Food Inspection Agency; Ontario Ministry of Agriculture, Food and Rural Affairs; Ontario Ministry of Health; Ontario Ministry of Natural Resources and Forestry; Public Health Agency of Canada; Public Health Ontario; Hospital, specialty clinic or private clinic; University, college or other educational institution; Other _____*
6. Length at current main employer: *less than 2 years; 2-5 years; 6-10 years; more than 10 years*

**Leptospirosis knowledge and risk perception**

1. How knowledgeable are you about leptospirosis in animals?: *Not knowledgeable at all; Slightly knowledgeable; Moderately knowledgeable; Very knowledgeable; Extremely knowledgeable*
2. How knowledgeable are you about leptospirosis in humans?: *Not knowledgeable at all; Slightly knowledgeable; Moderately knowledgeable; Very knowledgeable; Extremely knowledgeable*
3. Do you believe leptospirosis is a risk to public health in Ontario?: *Yes/No*
4. a) If yes, please briefly explain why*: _____ (response not required)*

b) If no, please briefly explain why not*: _____ (response not required)*

1. Which ONE of the following risk factors do you understand to contribute the most to cases of human leptospirosis in Ontario?: *Host (human) behaviour (e.g., time spent outdoors, occupation, etc); Contact with domestic or agricultural species; Geographic location, land use and/or habitat (e.g., living in an urban vs. rural location, proximity to water bodies, etc.); Contact with wildlife (including urban wildlife); Weather or climate (e.g., season, rainfall, temperature); Socio-demographic factors (e.g., housing, income, etc.); Other (please specify) _____*
2. Do you believe leptospirosis is a risk to domestic animal health in Ontario?: *Yes/No*
3. a) If yes, please briefly explain why*: _____ (response not required)*

b) If no, please briefly explain why not*: _____ (response not required)*

1. Which ONE of the following risk factors do you understand to contribute the most to cases of companion animal (i.e., pet) leptospirosis in Ontario? *Host (animal) behaviour (e.g., time spent outdoors, hunting dog, etc.); Contact with other domestic or agricultural species; Geographic location, land use and/or habitat (e.g., living in an urban vs. rural location, proximity to water bodies, etc.); Contact with wildlife (including urban wildlife); Weather or climate (e.g., season, rainfall, temperature); Socio-demographic factors (e.g., housing, income, etc.); Other (please specify) _____*
2. Which ONE of the following risk factors do you understand to contribute the most to cases of livestock (i.e., farm animal) leptospirosis in Ontario?: *Host (animal) behaviour (e.g., time spent outdoors, hunting dog, etc.); Contact with other domestic or agricultural species; Geographic location, land use and/or habitat (e.g., living in an urban vs. rural location, proximity to water bodies, etc.); Contact with wildlife (including urban wildlife); Weather or climate (e.g., season, rainfall, temperature); Socio-demographic factors (e.g., housing, income, etc.); Other (please specify) _____*
3. Do you believe leptospirosis negatively impacts wildlife health in Ontario?: *Yes/No*
4. a) If yes, please briefly explain why*: _____ (response not required)*

b) If no, please briefly explain why not*: _____ (response not required)*

1. In Ontario, what direct or indirect effects of leptospirosis do you feel are of greatest concern? Select up to 3: *Human clinical disease / illness; Human death / mortality; Human long term side effects of leptospirosis; Domestic and/or agricultural animal clinical disease; Domestic and/or agricultural animal death / mortality; Domestic and/or agricultural animal long term side effects of leptospirosis; Economic consequences (e.g., costs associated with ongoing surveillance and outbreak response, agricultural production loss, etc.); Wildlife illness or mortality; Other (please describe) _____*
2. Map: Districts of Ontario. Which areas of Ontario do you know, or suspect to be, an area of high leptospirosis activity? Please use the map to select any of the following nine regions in Ontario. If unsure, please advance to next question without making a selection *(Fig 4)*
3. Do you work with (or have the potential to work with) *Leptospira*/leptospirosis in any capacity in your current position? (e.g., handling biological samples, diagnostic testing, surveillance in any capacity, information sharing, patient interaction, research, etc.): *Yes/ No*

**Surveillance Perception**

1. Is current surveillance of leptospirosis adequate in Ontario?: *Yes for both animals and humans; Yes with respect to animals only; Yes with respect to humans only; No for both animals and humans*
2. If answered *Yes for both animals and humans, Yes with respect to animals only,* or *Yes with respect to humans only*: Is current surveillance of leptospirosis adequate in Ontario if leptospirosis prevalence and disease risk were to increase? *Yes/No*
3. Do you believe cross-sector data integration improves the understanding or surveillance of any disease/pathogen?: *Yes/No*

^a^ The complete survey included 43 questions. Only questions with responses analyzed in this paper are included in S1 File. The results from the remaining questions will be analyzed in future research. The full survey is available from the corresponding author upon request.
